# High Bacterial Diversity of Biological Soil Crusts in Water Tracks over Permafrost in the High Arctic Polar Desert

**DOI:** 10.1371/journal.pone.0071489

**Published:** 2013-08-13

**Authors:** Blaire Steven, Marie Lionard, Cheryl R. Kuske, Warwick F. Vincent

**Affiliations:** 1 Bioscience Division, Los Alamos National Laboratory, Los Alamos, New Mexico, United States of America; 2 Département de biologie, Université Laval, Québec City, Québec, Canada; 3 Centre d’Études Nordiques, Université Laval, Québec City, Québec, Canada; Dowling College, United States of America

## Abstract

In this study we report the bacterial diversity of biological soil crusts (biocrusts) inhabiting polar desert soils at the northern land limit of the Arctic polar region (83° 05 N). Employing pyrosequencing of bacterial 16S rRNA genes this study demonstrated that these biocrusts harbor diverse bacterial communities, often as diverse as temperate latitude communities. The effect of wetting pulses on the composition of communities was also determined by collecting samples from soils outside and inside of permafrost water tracks, hill slope flow paths that drain permafrost-affected soils. The intermittent flow regime in the water tracks was correlated with altered relative abundance of phylum level taxonomic bins in the bacterial communities, but the alterations varied between individual sampling sites. Bacteria related to the Cyanobacteria and Acidobacteria demonstrated shifts in relative abundance based on their location either inside or outside of the water tracks. Among cyanobacterial sequences, the proportion of sequences belonging to the family Oscillatoriales consistently increased in relative abundance in the samples from inside the water tracks compared to those outside. Acidobacteria showed responses to wetting pulses in the water tracks, increasing in abundance at one site and decreasing at the other two sites. Subdivision 4 acidobacterial sequences tended to follow the trends in the total Acidobacteria relative abundance, suggesting these organisms were largely responsible for the changes observed in the Acidobacteria. Taken together, these data suggest that the bacterial communities of these high latitude polar biocrusts are diverse but do not show a consensus response to intermittent flow in water tracks over high Arctic permafrost.

## Introduction

Polar soils underlain by permafrost display unique drainage patterns. One such pattern is the formation of water tracks. As water cannot penetrate the ice table (the upper boundary of permafrost) moisture can route downstream through the active layer resulting in zones of high soil moisture surrounded by relatively drier soils [1]. Water tracks have been shown to alter nutrient flow and increase plant productivity in tundra soils [2], [3], and in the Antarctic polar desert they have been referred to as “salt superhighways” in which there are elevated rates of soil weathering and biogeochemical activity [4]. The pulsed water flow in these water tracks results in characteristic landscape deformations, although soil moisture in the water tracks can vary widely and is tied to local environmental factors such as snow melt [5]. In this regard, the water tracks represent a micro-environment in which intermittent periods of water flow may foster localized changes in biological community structure and activity. Despite the importance of water tracks to polar hydrology and ecology, little is known regarding how bacterial communities vary due to the water pulses present in permafrost water tracks.

**Figure 1:**
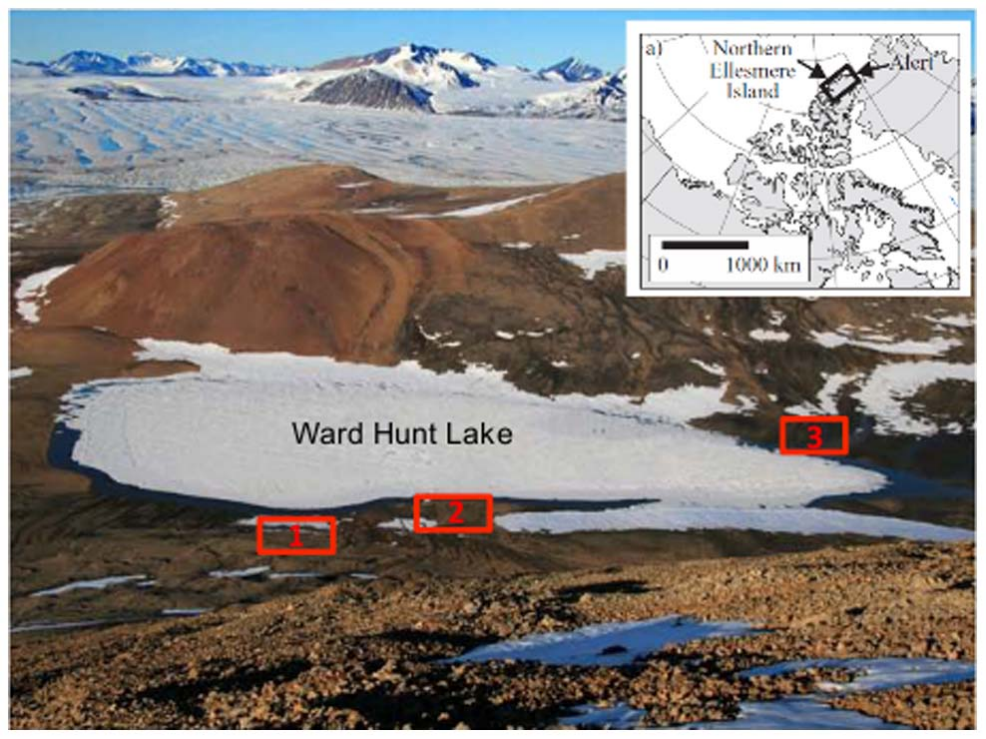
Ward Hunt Island. The three sampling sites around Ward Hunt Lake are shown in the red boxes. The inset map displays the location of Ward Hunt Island in the Canadian Arctic archipelago. Global positioning system coordinates for the sampling sites: Site 1, 83°05.263’N, 74°10.293’W; Site 2, 83°05.268’N, 74°10.346’W; Site 3, 83°04.887’N, 74°10.491’W.

Ward Hunt Island is located 6 km off the northern tip of Canada (Figure 1). The mean annual temperature of the Ward Hunt Island region is −15.9°C and mean annual precipitation is 154 mm, making this region an extreme polar desert [6], although less arid than analogous environments in Antarctica [1]. Previous studies at Ward Hunt Island have characterized the microbial communities in the local ice shelves [7], snow pack [8], and the microbial mats from Ward Hunt Lake (the drainage catchment for the water tracks in this study) have previously been studied by pigment and molecular methods (e.g. [9], [10]). However, none of these studies have characterized the biocrusts that inhabit the soils surrounding the lake or the water tracks that may serve as a connection between the local soils and the lake environment. Furthermore, water limitation has been found to be the dominant factor determining the distribution and abundance of biocrusts in polar desert soils [11], so we hypothesized that water tracks would significantly alter the structure and composition of the local soil bacterial communities.

In this study, we employed replicated high-throughput bacterial 16S rRNA gene sequence analysis to characterize the bacterial communities in Ward Hunt Island biocrusts and to investigate the effect of intermittent flow in permafrost water tracks on the structure of the indigenous bacterial populations, both within the tracks and in the adjacent soils.

## Materials and Methods

### Sample Collection

Soil samples were collected from the catchment area of Ward Hunt Lake. Ward Hunt Lake (83°05.297’N, 74°09.985’W) is an ultra-oligotrophic water body situated on Ward Hunt Island, off the northern coast of Ellesmere Island in the Canadian High Arctic (Figure 1). Any snow typically begins to melt in late May-June, with runnels of flowing water (water tracks) that discharge to the eastern and western sides of the lake. Soil samples were collected on July 8, 2011 at three sites around Ward Hunt Lake (Figure 1): two on the western part of the lake at the base of Walker Hill (sites 1 and 2), and one site on the eastern side of the lake (site 3). Site 1 was a relatively dry zone with clear visible water tracks, which were moist at the time of sampling. Site 2 was generally wetter with clear formation of water tracks. Site 3 was also in a dry zone with well delimited water tracks. The tracks were generally 10 to 20 cm wide and were darker in surface coloration relative to the ≥2 m of slightly higher ground that separated them. At each site, two samples were collected representing soils inside and outside of the water tracks.

Soil samples were collected with a sterile sectioned 50 ml syringe to a depth of 1 to 1.5 cm. Because the water content of the soils varied, the weight of the soils differed between samples. Soils were stored locally in a solar powered freezer at −20°C and returned to Centre d’Ètudes Nordiques (CEN), Québec, Canada. For sequencing, the samples were shipped overnight to Los Alamos National Laboratory, NM USA on dry ice. Soils at each site were primarily composed of gravel and large pebbles were removed prior to chemical characterization or DNA extraction.

Samples were collected under the Parks Canada Agency, Research and Collection Permit QUT-2011-8555 and the Nunavut Research Institute (NRI) Science License 02 125 11 N-M.

### Soil Characterization

Determination of the elemental composition of samples was conducted at CEN. Water content was determined as loss of mass after 48 hours of drying at 70°C. Samples were then ground and 100 to 180 mg of soil was burned at 1000°C in an excess of oxygen using a LECO CHN 628 (Leco Corporation, St. Joseph, MI) to determine carbon, and nitrogen.

### DNA Extraction and 16S rRNA Gene Sequencing

Frozen soil cores were sectioned at −20°C to exclude the outer *ca.* 1 cm of soil material, to avoid potential sampling contaminants, as previously described [12]. Total community DNA was extracted in a sterile laminar flow hood via bead beating lysis using the MoBio PowerMax Soil DNA Isolation Kit (MoBio Laboratories, Inc. Carlsbad, CA) and supplied protocols. After extraction, DNA concentrations were normalized to 1 ng µl^-1^. The 16S rRNA genes were amplified using primers targeting the V5 and V6 regions as described by Claesson *et al*. [13]. Eight base pair barcodes for multiplex sequencing were designed using the BARCRAWL software [14] and were added to the reverse primer. Amplification of 16S rRNA genes was performed using reaction concentrations and thermocycling conditions described previously [15]. Briefly, thermocycling consisted of an initial denaturation of 95°C for 3 min, followed by 30 cycles of 95°C for 30 s, 54°C for 30 s, and 72°C for 30 s, cycling was followed by a final extension of 72°C for 10 min. Successful amplification was verified by gel electrophoresis and amplification products were sequenced via 454 FLX Titanium using standard protocols [16]. Each technical PCR replicate represents an individual PCR reaction (including a unique barcode) generated from the sample DNA template. Therefore each site was represented by three independent technical PCR replicates from both inside and outside water tracks.

### Sequence Analysis and Statistical Comparisons

Raw sequences were processed using the mothur software [17] implementation of PyroNoise [18] and selecting sequences with no ambiguous bases and no homopolymers of >8 bp. Sequences were aligned against the SILVA [19] reference alignment as implemented in mothur and sequences that did not align or did not align over the expected region of the alignment were discarded. Potential chimeric sequences were detected with the UCHIME algorithm [20] and identified chimeras were also discarded.

Sequences were clustered into operational taxonomic units (OTUs) using average neighbor clustering and diversity statistics (non-parametric Shannon’s diversity, Shannon’s evenness, and Good’s coverage [21]) were calculated within the mothur package. Statistically significant differences in diversity metrics were assessed using an ANOVA with a Tukey’s HSD mean separation at p<0.05 using the JMP Statistical Discovery Software version 5.1 (SAS, Cary, NC). As PCR replicates were not found to differ significantly, comparisons among the different sites and inside and outside of water track samples were made between averaged technical replicate values. Pearson’s correlations between soil characteristics or between soil characteristics and bacterial relative abundance were performed with the Free Statistics Software [22].

Principal coordinates analysis was determined using OTU membership and abundance. Comparisons based on community composition were determined using the Jaccard index and comparisons based on community structure employed the abundance-based Jaccard index, calculated in the mother software using the 97% sequence identity OTUs.

For the determination of the identity of the dominant OTU representative sequences, the datasets were compiled so that the representative OTUs are the numerically abundant OTUs across the technical replicates and sites. The representative sequences were culled from the 97% OTU data using weighted abundance to ensure representative sequences were the most abundant sequence type among each OTU. Representative sequences from the 25 most abundant OTUs were selected for analysis to limit possible inflation of sequence novelty due to rare species or potential sequencing error. Representative sequences were compared to previously described 16S rRNA sequences using a BLASTn query against GenBank [23]. Taxonomic classification of sequence reads was performed using the Ribosomal Database project taxonomy [24].

### Sequence Dataset Availability

All sequence datasets and corresponding metadata have been made publically available through MG-RAST [25] under the project “Ward Hunt water tracks” (MG-RAST identification numbers: 4519154.3–4519171.3).

## Results

### Soil Characterization

Nutrient levels of soils collected from inside and outside of the respective water tracks are shown in Table 1. Note that at the time of sampling two water tracks were drier than the soils outside the water tracks, and all of the soils were relatively moist as a result of the recent snow melting (Table 1). A Pearson product-moment correlation coefficient was computed to assess the relationship between water content and soil nutrients. Water content was positively correlated with carbon (r = 0.822, p = 0.044) and nitrogen (r = 0.912, p = 0.010), suggesting that the nutrient status of these soils is tied to the soil water status at the time of sampling, independent of whether or not the samples were taken from inside or outside of the water tracks.

**Table 1:** Characteristics of soil samples.

Site	Location in water tracks	Water content (%)	Nitrogen (%)	Carbon (%)
Site 1	Outside	41	0.70	11.67
Site 1	Inside	59	1.02	15.61
Site 2	Outside	58	0.85	18.97
Site 2	Inside	48	0.54	8.67
Site 3	Outside	41	0.34	4.25
Site 3	Inside	23	0.08	4.65

### Sequence Numbers and Diversity of 16S rRNA Datasets

Sequencing and diversity statistics for each dataset are displayed in Table 2. A total of 135,358 16S rRNA sequences were generated consisting of >20,000 OTUs defined as sequences sharing >97% sequence identity. A more conservative estimate of the number of OTUs, obtained by removing singleton OTUs (OTUs represented by a single sequence), indicated that across the samples 7432 OTUs were identified (1496–1857 in individual datasets).

**Table 2:** Sequencing and diversity statistics^a^.

Site	Location	Nseqs	OTUs^b^	H’^b^	Evenness^b^	Coverage^b^
Site 1	Outside	7957	2116(1818)^c^	6.94	0.87	74.3
Site 1	Inside	7367	1801(1496)	6.37	0.80	78.0
Site 2	Outside	7418	2167(1803)	7.06	0.88	73.7
Site 2	Inside	7720	1801(1572)	6.31	0.79	78.1
Site 3	Outside	6730	2265(1786)	7.16	0.89	72.6
Site 3	Inside	7926	2246(1857)	7.09	0.87	72.3
**Total** d		**135358**	**21460** **(7432)**	**7.75**	**0.76**	**89.6**

a Values for the sites represent averages across the 3 technical replicates.

b Calculated with an OTU definition of 97% sequence identity and datasets randomly subsampled to the size of the smallest dataset (5609) sequences.

c Number of OTUs after singletons (OTUs made up of 1 sequence) were removed.

d Calculated with all sequences compiled.

Comparison of means statistics indicated that there were no significant differences in diversity among technical replicates or between the three sites sampled from the same location relative to the water tracks (inside or outside Table 2). Comparatively, diversity tended to be higher in the samples outside of the water tracks, both in the number of recovered OTUs and in Shannon’s diversity index (H’), with p-values of 0.10 and 0.07, respectively. Shannon’s evenness was significantly higher in the outside samples (p = 0.05) while there was no significant difference in coverage (Table 2).

### Comparisons to Other Temperate Latitude and Arctic Environments

The number of OTUs recovered and Shannon’s diversity indices of these datasets were compared to similar datasets generated from selected environments (Table 3). The environments represented were cyanobacterial mats, cyanobacteria-dominated systems similar to biocrusts; soils, as the biocrusts are a soil environment; and previous Ward Hunt Island studies, as these sites are from sites from the same locality of the current study (Table 3). These high Arctic biocrust communities were as, or more diverse, than other environments from more temperate locations, such as grassland soils, agricultural soils, and other cyanobacterial biocrusts (Table 3). These biocrusts surrounding Ward Hunt Lake also appeared to be more diverse than other Ward Hunt Island environments (Table 3). It should be noted, however, that this study represents the first use of next-generation sequencing to describe the bacterial diversity in these Ward Hunt Island soils and the scale of sequencing was orders of magnitude higher than in most of the previous studies conducted at Ward Hunt Island (Table 3).

**Table 3:** Comparison of bacterial diversity of these datasets to selected environments.

Study site	Sequencingregion^a^	AverageSequences	OTUs (% sequence identity)	H’	Reference
***Cyanobacterial mats and crusts***					
Saline lake sediments, Australia	V1–V2	12981	640–1422 (97%)	NA^b^	[51]
Soil crusts, USA	V5–V6	3236	367–408 (97%)	6.4–6.8	[52]
Coastal mats, Netherlands	V5–V6	31044	710–2593 (95%)	5.1–6.3	[53]
Thrombolitic mats, Bahamas	V1–V2	1992	295–348 (97%)	5.2–5.5	[54]
***Soil***					
Agricultural soil, pH gradient, United Kingdom	V1–V2	600	198–400 (97%)	NA	[42]
Grassland soil, Germany	V2–V3	41824	4781–6231 (97%)	5.6–7.1	[55]
Oak rhizosphere and bulk soil	V5–V6	50314	4886–8281	6.8–7.5	[56]
Pampas forest and grasslands, Brazil	V2–V3	21256	142–172 (polygenetic distance)	10.0–10.6	[57]
Rhizosphere, Antarctica	V5–V6	2000	552–732 (97%)	4.9–5.7	[58]
***Ward Hunt environments***					
Ice shelves	V1–V5	159	52–105 (98%)	3.5–4.4	[7]
Snow pack	V1–V5	57	10–18 (97%)	0.2–1.9	[8]
Lake benthic mat cyanobacteria	V2–V5	71	5–12 (97%)	0.7–2.0	[9], [10]
Meromictic lake	V6–V8	6684	280–425 (97%)	NA	[59]
**This study**	**V5**–**V6**	**7520**	**1801**–**2264 (97%)**	**6.4**–**7.2**	

a Sequencing region defined as the variable regions included in the sequence.

b Not available.

The 25 most abundant OTUs (sequence bins containing the largest number of sequences) were culled from the pooled dataset and classified via BLAST. All of the OTUs were closely related or identical to previously characterized 16S rRNA sequences (Table 4). The location from which the closest matched 16S rRNA sequences were isolated encompassed diverse habitats, including Arctic, alpine, Antarctic, soil, and non-soil environments (Table 4). These data suggest that species closely related to those in these polar biocrusts are common to many different environments.

**Table 4:** Similarity of representative sequences from the 25 most abundant OTUs to sequences in GenBank.

Number of sequences	% sequence identity	Isolation source	Accession number	Taxonomy
5087	99	Loess Plateau, China	JN559117.1	Acidobacteria
3237	99	Rock biofilm	FM253596.1	Verrucomicrobia
3101	100	Sequestered chloroplast	HM213811.1	chloroplast
2805	99	Apple orchard	KC331596.1	Acidobacteria
2182	99	Alpine grassland	JQ825224.1	Verrucomicrobia
2047	99	Soil crust	JX255264.1	Cyanobacteria
1730	99	Polar microbial mat	JX887889.1	Cyanobacteria
1677	99	Arctic snow	FJ946535.1	chloroplast
1506	99	Subway aerosol	JX395784.1	chloroplast
1391	100	Acidic stream	JQ815669.1	chloroplast
1231	99	Dry Valley stream	EU869771.1	Acidobacteria
1089	99	NA	DQ629514.1	chloroplast
1013	99	Loess Plateau, China	JN559144.1	unclassified
794	99	Mesotrophic lake	JQ942119.1	Verrucomicrobia
761	99	PAH contaminated soil	FQ660498.1	Verrucomicrobia
733	99	Loess Plateau, China	JN559163.1	Proteobacteria
728	99	Rhizosphere soil	JX489893.1	Acidobacteria
714	99	Showerhead biofilm	EU631953.1	Verrucomicrobia
707	99	Arctic snow	FJ946535.1	chloroplast
698	99	Greenland ice sheet	JQ407495.1	Cyanobacteria
643	99	Alkaline lake	JN825402.1	Proteobacteria
606	100	Lava tube	JF265892.1	Acidobacteria
602	99	Alpine grassland	JQ825192.1	Planctomycetes
539	100	Himalayan glacier	GQ366692.1	Actinobacteria
501	99	Human microbiome	GQ002624.1	chloroplast

### Relationship between Technical Replicates, Sites, and Position Inside or Outside of Water Tracks

**Figure 2:**
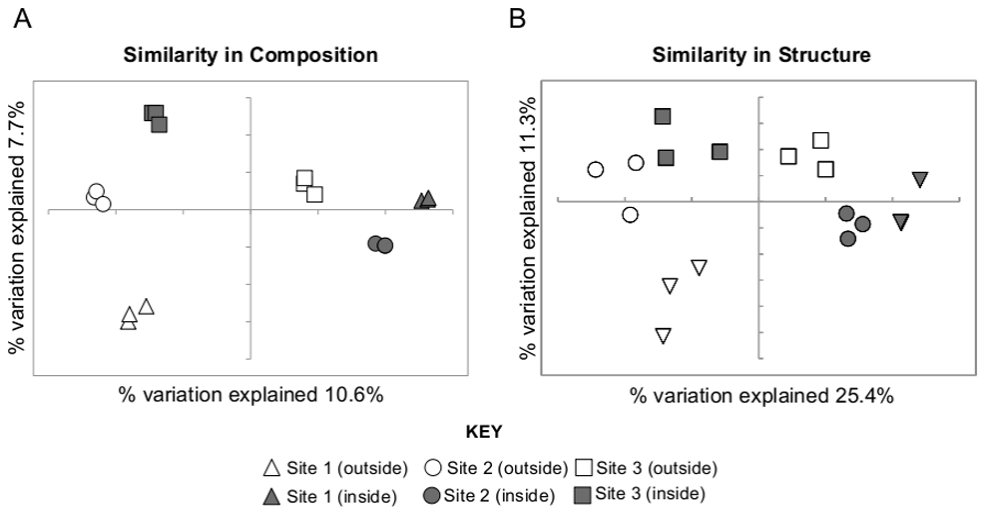
Principal component analysis of the relationship between 16S rRNA gene sequence datasets. A) Clustering of datasets based on community membership. B) Clustering of datasets based on community structure.

Figure 2A displays similarities in community composition between the different datasets. Technical replicates clearly clustered, demonstrating that the independent sequencing reactions recovered similar community members. Each sample clustered independently with no apparent clustering of sites or clustering due to the position inside or outside of the water tracks (Figure 1). ANOSIM variance statistical testing indicated the differences in community membership and structure were consistently significant with p-values of <0.001, both between sites and between samples taken from inside or outside of the water tracks at the same site. Together these data indicate that each site harbors a distinct bacterial community (Figure 2A).

The datasets were also clustered based on community structure, incorporating relative abundance of OTUs (Figure 2B). The technical replicates still clustered, although the dissimilarity (distance between datasets) was larger, indicating that differences in replicates are primarily due to differences in the abundance of sequences rather than in the sequences recovered. Overall, clustering of datasets showed a similar relationship between the different sites, and position inside or outside of the water tracks, indicating that the observed community differences were similar when considering both community membership and structure (Figure 2).

### Taxonomic Composition of Sequence Datasets

**Figure 3:**
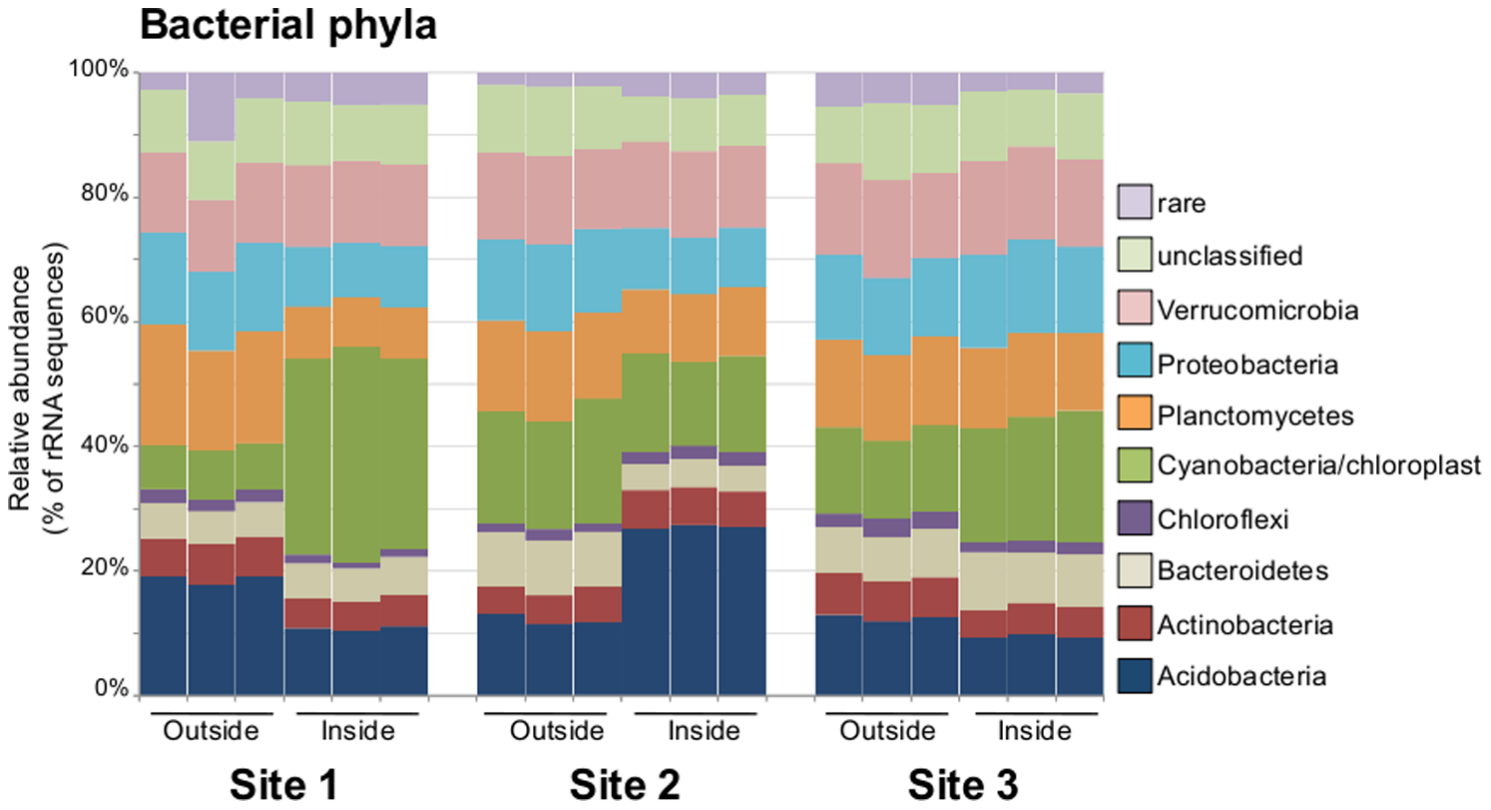
Relative abundance of phylum level taxonomic bins in the 16S rRNA gene sequence datasets. The 10 most abundant phyla across the datasets are displayed. The category rare represents the sum total of all phyla made up of <1% of sequences.

Across the different samples 29 bacterial phyla were identified (Supplementary Table S1). The ten most abundant phyla accounted for >90% of the recovered sequences across all of the samples. The relative abundances of the 10 dominant phyla are displayed in Figure 3. Over 50% of the sequences in most samples were made up of sequences related to just five phyla: Acidobacteria, Cyanobacteria, Proteobacteria, Planctomycetes, and Verrucomicrobia (Figure 3).

**Figure 4:**
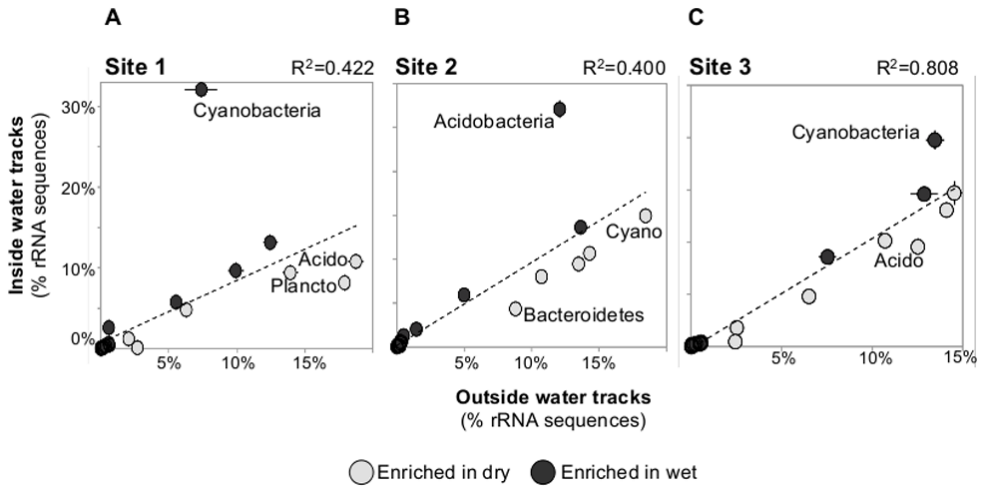
Correspondence between phyla relative abundance inside and outside of water track samples. Each phylum detected is represented by a point in the graph and the points are colored based on whether they are more abundant in the inside or outside samples. Error bars represent the standard deviation of the mean across technical replicates (in most cases error bars do not extend outside of the plotted point). Points showing large differences in abundance between inside and outside of the water tracks are labeled with the phylum name. Abbreviations: Acido, Acidobacteria; Cyano, Cyanobacteria; Plancto, Planctomycetes.

The original study design involved collecting three field replicates inside and outside of the water tracks with technical replicates for each sample. Given the lack of consensus response to wetting pulses inside the water tracks, in both soil chemistry (Table 1) and taxonomic composition (Figure 2, 3), each site was investigated separately for effects of intermittent water pulses. At each site, the relative abundance of phylum level bins was plotted to test the correspondence of phyla abundance between the inside and outside of water track samples, and to identify phyla that show large differences in relative abundance based on their position either inside or outside of the water tracks (Figure 4). At Site 1, Cyanobacteria were more abundant in the inside water track samples whereas Acidobacteria and Planctomycetes accounted for a higher proportion of the outside samples (Figure 4A). Comparatively, Site 2 showed an increase of Acidobacteria in the inside samples with a concurrent increase in the proportional representation of Bacteroidetes in the outside samples (Figure 4B). At Site 3, the responsive taxa were similar to Site 1, showing an increase in the relative abundance of Cyanobacteria in inside samples and an increase of Acidobacteria in the outside samples (Figure 4C), although the magnitude of change in relative abundance was lower than observed at Site 1. The correspondence between the outside and inside samples was higher at site 3 than at the other two sites (Figure 4), suggesting the alterations in the community composition related to position in the water tracks was less pronounced at Site 3. These data suggest that there was not a consensus response of the bacterial communities due to their position in the water tracks.

As the phyla Acidobacteria and Cyanobacteria appeared to be the most responsive to changes in relative abundance based on their position inside or outside of the water tracks (Figure 4) the local soil factors inside and outside the water tracks (Table 1) were tested for a correlation with the relative abundance of Acidobacteria or Cyanobacteria. There was not a significant correlation of the relative abundance of Acidobacteria to any of the measured soil characteristics. Similarly, Cyanobacteria relative abundance did not correlate with any of the soil characteristics.

**Figure 5:**
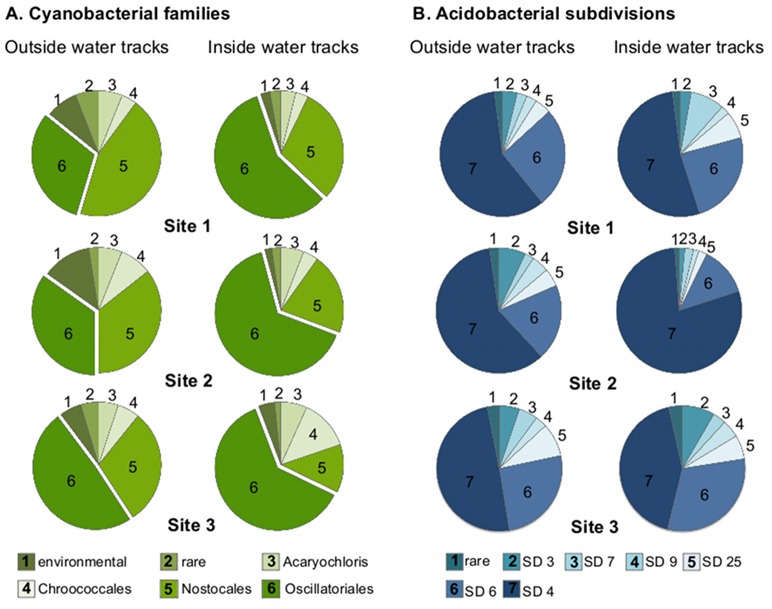
Taxonomic composition of cyanobacterial and acidobacterial populations. A) Family level taxonomic bins within the cyanobacterial sequences. The category environmental represents sequences most closely related to uncharacterized sequences from environmental surveys. B) Subdivision (SD) level taxonomic bins within the acidobacterial sequences. The category rare represents the sum total of subdivisions that individually accounted for <1% of acidobacterial sequences.

To better define the organisms that may be driving the differences in relative abundance of these phyla, they were investigated at deeper taxonomic levels. Across the samples *ca.* 90% of Cyanobacteria sequences could be classified to the family level (Figure 5A). Among the Cyanobacteria, at all three sites, the proportion of Oscillatoriales related sequences was higher in the samples from inside the water tracks while there was generally a decrease in the proportion of Nostocales and environmental cyanobacteria-related sequences (Figure 5A).

The majority (>90%) of Acidobacteria-related sequences belonged to six subdivisions (Figure 5B). Site 2 showed the largest increase in Acidobacteria-related sequences inside the water tracks (Figure 3), and an increase in the proportion of subdivision 4 Acidobacteria inside the water tracks (Figure 5B). Comparatively, sites 1 and 3 showed a decrease of total Acidobacteria-related sequences and a concurrent decrease in the proportion of subdivision 4 Acidobacteria (Figure 5B) suggesting that changes in the abundance of the subdivision 4 Acidobacteria may be largely responsible for the acidobacterial phylum level differences in relative abundance.

## Discussion

### Diversity of Ward Hunt Island Biocrusts

This study adds to a growing body of literature that documents high bacterial diversity in Arctic soils [26], [27]. We hypothesized that the bacteria in biocrusts on Ward Hunt Island would be limited in diversity as these soils are characteristic of an extreme polar desert and occur at the northern limits of soil ecosystems. Contrary to our expectations the Ward Hunt biocrust bacterial communities were diverse, often as diverse as analogous environments from more temperate systems (Table 3). These results support previous observations that suggest latitudinal species sorting is not a significant factor in soil microbial community assembly [28]. Although the present study did not encompass comparative analyses across a latitudinal gradient, the sampling site analyzed here can be viewed as the northernmost soil environment (along with northern Greenland) that could be sampled in any latitudinal study.

At the southern limit of soil ecosystems in the Antarctic, relatively low diversity of soil microbial communities is common [e.g. 29,30,31], although detailed microbiological analyses are required of water track communities in the south polar environment. Clone library analyses of moist soils near flowing streams in Antarctica have revealed a higher microbial diversity than in dry soils [32]. Somewhat paradoxically, while Arctic bacterial communities tend to be more diverse than analogous Antarctic communities, similar or identical bacterial OTUs are often found in both poles. For example, freshwater bacterioplankton with identical phylogenetic gene markers have been detected in terrestrial Arctic and Antarctic lakes [10], [33]. Here we show that the dominant soil OTUs are identical or closely related to sequences recovered from a broad diversity of environments, including Antarctic Dry Valley soils (Table 4). Increased diversity of the Arctic microbial communities suggest that dispersal, colonization, or establishment mechanisms differ between the two polar regions of planet Earth. However, shared bacterial species between these high Arctic biocrusts and other polar environments implies certain microbial species, well adapted for survival in the cold biosphere, can achieve bi-polar distributions. In the Arctic, terrestrial systems are generally continuous to the northern extremes, separated by relatively small water channels, or are seasonally bridged by sea ice. In comparison, the Antarctic is geographically isolated. With the exception of the western Antarctic Peninsula, terrestrial Antarctic soil environments are segregated from other land masses by large expanses of the Southern Ocean. Furthermore, the few patches of exposed soil on the Antarctic continent occur in the coastal regions and are divided by the continental ice-sheets [34]. The increased connectivity among Arctic terrestrial environments may partially explain the observations of diverse bacterial ecosystems in the Arctic compared to relatively species poor populations in the Antarctic. In this regard, the Earth’s poles are a unique natural laboratory in which to test the dispersal and survival limitations of microbial life.

It is important to note that the community profiling used in this study was based on environmental DNA so would detect active, dormant, and even possibly dead preserved cells or DNA. Given the cold desiccating conditions of Arctic soils, it has been proposed that these soils may harbor substantial populations of quiescent cells or exogenous DNA [35], [36], [37], [38], [39]. Yet, the data presented here suggest that the intermittent flow in water tracks resulted in shifts in the microbial community, potentially due to the growth of specific lineages of bacteria related to the Oscillatoriales-cyanobacteria and subdivision 4 Acidobacteria (Figure 5). These data suggest that at least a sub-population of the polar desert bacterial communities may be viable and can respond to elevated water supply that occurs in pulses within the water tracks. Future studies employing techniques such as stable isotope probing [40] or RNA sequencing will need to be employed to specifically identify and characterize any active microbial populations.

### Effects of Position Relative to Water Tracks on Bacterial Communities

Several studies have documented that local environmental factors, such as pH, soil moisture, and land use history exert stronger influences on microbial communities than climatic, elevation or latitudinal gradients [28], [41], [42], [43], [44]. In desert soils microbial diversity and activity are primarily limited by water potential [45]. Additionally, changes in hydrology are considered to be one of the largest factors driving changes in Arctic systems [46]. In this study, intermittent wetting in water tracks over permafrost soils was used to test the effects of a localized environmental perturbation on the indigenous bacterial communities. At the phylum level a consensus response to location relative to the water tracks was not apparent. For example, the proportion of cyanobacteria-related sequences increased inside water tracks at sites 1 and 3 but decreased slightly inside the water tracks at site 2 (Figure 4). The soils in all of the sites in the present study were relatively moist, associated with the recent snow melt and possibly wicking from the water tracks, and this may be a factor contributing to the relatively small and inconsistent differences in community structure associated with samples from inside and outside of the water tracks. However, at the family level Oscillatoriales-related cyanobacteria consistently increased inside the water tracks (Figure 5A), suggesting specific cyanobacteria may have responded to the pulsed flow. Oscillatoriales-related cyanobacteria are the dominant biocrust forming cyanobacteria in dryland biocrusts [47], [48] and previous work in arid soils has shown that oscillatorian cyanobacteria are tactile towards water [49]. The apparent enrichment of oscillatorian cyanobacteria inside the water tracks may suggest more developed biocrusts [50]. Linking changes in the cyanobacterial community to biogeochemical cycle rates may indicate that water tracks in polar deserts are hot spots for primary productivity, similar to what has been described for plant populations [2].

Another trend observed in the bacterial communities in association with the water tracks was an alteration of the proportion of bacteria of the phylum Acidobacteria. Particularly subdivision 4 acidobacterial sequences seemed to track the changes in the total relative abundance of Acidobacteria (Figure 5). Previous studies have documented that Acidobacteria are among the most responsive populations in tundra soil under snow packs, suggesting that Acidobacteria are potentially reactive to water inputs [45].

Water tracks in permafrost soils represent an avenue for microbial emigration or nutrient transport from soil to local freshwater systems. In recent history, Ward Hunt Lake has remained perennially ice covered [6], but in the summers of 2011 and 2012 the lake ice completely melted (M. Paquette *et al*. unpublished observations). These warm ice-free periods increase the potential for local soil organisms to inoculate the lake through increased flow in water tracks or aerosol deposition. Tracking the development of microbial communities in the vicinity of Ward Hunt Lake will document the effects of opening a previously ice-covered ecosystem and increasing its connection to local microbial populations. Going forward, understanding how freshwater lakes and local soil environments are connected and how these connections transform under environmental perturbations will be essential to predicting how these systems will respond to a changing climate.

## Supporting Information

Table S1 **Bacterial phyla represented at the different sites inside and outside of water tracks.**
(DOC)
