# Supplementary material for: High Bacterial Diversity of Biological Soil Crusts in Water Tracks over Permafrost in the High Arctic Polar Desert
Source: PLoS One. 2013 Aug 13;8(8):e71489. doi: 10.1371/journal.pone.0071489 (PMC3742766; doi:10.1371/journal.pone.0071489)
Supplement: Table S1 — Bacterial phyla represented at the different sites inside and outside of water tracks. (DOC) [file pone.0071489.s001.doc]

| **Phyla** | **Site 1^a^** |  | **Site 2^a^** |  | **Site 3^a^** |  |
| --- | --- | --- | --- | --- | --- | --- |
|  | **Outside** | **Inside** | **Outside** | **Inside** | **Outside** | **Inside** |
| Acidobacteria | 20.22 | 11.77 | 13.66 | 28.44 | 13.86 | 10.87 |
| Actinobacteria | 6.83 | 5.13 | 5.42 | 6.24 | 7.13 | 5.04 |
| Aquificae | 0.01 | 0.03 | 0 | 0.01 | 0.03 | 0.04 |
| Armatimonadetes | 0.25 | 0.34 | 0.37 | 0.21 | 0.37 | 0.30 |
| Bacteroidetes | 5.65 | 5.86 | 8.96 | 4.49 | 7.70 | 8.91 |
| BRC1 | 0.06 | 0.03 | 0.05 | 0.03 | 0.08 | 0.03 |
| Chlamydiae | 0.25 | 0.14 | 0.26 | 0.13 | 0.16 | 0.23 |
| Chlorobi | 0.21 | 0.21 | 0.20 | 0.16 | 0.23 | 0.26 |
| Chloroflexi | 1.93 | 1.58 | 1.49 | 1.87 | 2.27 | 1.78 |
| Chrysiogenetes | 0.01 | 0.02 | 0.01 | 0 | 0.01 | 0 |
| Cyanobacteria/chloroplast | 7.50 | 33.1 | 19.01 | 15.12 | 13.28 | 19.77 |
| Deferribacteres | 0 | 0.01 | 0.02 | 0.02 | 0.02 | 0.01 |
| Deinococcus-Thermus | 0.02 | 0.03 | 0.05 | 0.03 | 0.04 | 0.03 |
| Elusimicrobia | 0.17 | 0.08 | 0.07 | 0.12 | 0.08 | 0.06 |
| Firmicutes | 8.30 | 3.99 | 3.67 | 4.93 | 5.61 | 4.50 |
| Fusobacteria | 0.02 | 0.07 | 0.01 | 0.05 | 0.05 | 0.02 |
| Gemmatimonadetes | 0.59 | 2.72 | 0.42 | 1.37 | 2.46 | 0.49 |
| Lentisphaerae | 0 | 0 | 0 | 0 | 0.01 | 0 |
| Nitrospira | 0.41 | 0.35 | 0.38 | 0.56 | 0.51 | 0.40 |
| OD1 | 2.41 | 2.36 | 1.07 | 1.20 | 1.73 | 1.34 |
| Planctomycetes | 15.20 | 6.52 | 13.36 | 8.50 | 12.50 | 12.37 |
| Proteobacteria | 16.32 | 11.12 | 16.99 | 11.31 | 15.60 | 17.41 |
| Spirochaetes | 0 | 0.14 | 0.03 | 0.12 | 0.03 | 0.01 |
| SR1 | 0.02 | 0 | 0.01 | 0.01 | 0.01 | 0.04 |
| Synergistes | 0.04 | 0.02 | 0.06 | 0.03 | 0.06 | 0.03 |
| Thermotogae | 0.02 | 0.01 | 0.03 | 0.03 | 0.01 | 0.06 |
| TM7 | 0.69 | 0.73 | 0.34 | 0.71 | 0.74 | 0.71 |
| Verrucomicrobia | 12.62 | 13.28 | 13.72 | 13.89 | 14.84 | 14.93 |

**^a^** Values represent averages across the technical replicates
